# Biomechanical effects of steroid injections used to treat pyogenic flexor tenosynovitis

**DOI:** 10.1186/1749-799X-7-34

**Published:** 2012-10-09

**Authors:** Blake R Turvey, Paul S Weinhold, Reid W Draeger, Donald K Bynum, Laurence E Dahners

**Affiliations:** 1 University of North Carolina, School of Medicine, Department of Orthopaedics, Campus Box #7055, Bioinformatics Building, Chapel Hill, NC 27599-7055, USA

**Keywords:** Pyogenic flexor tenosynovitis, Corticosteroids, Tensile strength, Biomechanical properties, Closed-space infection

## Abstract

**Background:**

A recent study from our laboratory has demonstrated improved range of motion in the toes of broiler chickens afflicted with pyogenic flexor tenosynovitis when treated with local antibiotic and corticosteroid injections, without surgical drainage. However, the use of corticosteroids as an adjunct treatment raised peer concern, as steroids are thought to have deleterious effects on tendon strength. The purpose of this study was to compare the tensile strength of the aforementioned steroid treated tendons, to a group of tendons administered with the current standard treatment: systemic antibiotics, surgical drainage and no corticosteroids.

**Methods:**

Twenty-three tendons’ structural and material properties were investigated (fifteen receiving the standard treatment, eight receiving the steroid treatment). The measurements from each group were interpreted via Student’s unpaired t-test and a post-hoc power analysis.

**Results:**

The steroid treated tendons did demonstrate a trend toward decreased mechanical properties when compared with the standard treatment group, but the results were not statistically significant.

**Conclusions:**

Treatment of septic tenosynovitis with local corticosteroid and local antibiotic injections resulted in better digital motion, without a significant loss of tendon strength, over a twenty-eight day recovery period.

## Background

Pyogenic flexor tenosynovitis (PFT) is a serious closed space infection of the digital flexor tendon sheath, most commonly caused by *Staphylococcus aureus*[1-3]. If not properly treated, the infection may result in significant morbidity, including loss of motion or ultimately amputation [4,5]. Currently, the standard treatment for this infection involves surgical drainage of the infection and an appropriately tailored systemic antibiotic therapy without adjunct steroids.

A prior study performed by Draeger et al. in our laboratory demonstrated improved range of motion with the addition of corticosteroids to antibiotics, with or without surgical drainage, as a treatment regimen for PFT in a broiler chicken digital septic tenosynovitis model [6]. However, concern has been expressed that exposure of tendons to corticosteroids compromises their structural integrity and might lead to an increased rate of tendon rupture [7-10]. Due to these concerns, we felt it prudent to further investigate the effects of peritendinous corticosteroid injections on the tendon’s structural and material properties in this condition.

There is evidence suggesting that intratendinous corticosteroid injections temporarily decrease tendon strength 3–4 weeks following injections [11,12]. However, the Draeger study administered dexamethasone into the tendon sheath, where similar corticosteroid injections have been shown to beneficially act as anti-inflammatory agents [13,14].

Because the feet of the chickens used in the study conducted by Draeger were frozen and preserved, we had an opportunity to evaluate the mechanical and structural properties of the tendons in response to the questions raised. Draeger demonstrated that a local antibiotic and steroid injected group treated without surgical drainage retained significantly better motion than a systemic antibiotic and surgical drainage group (the current standard treatment) at all post-treatment time points [6]. Therefore, the primary focus of this study was to compare the biomechanical strength between these two groups.

## Methods

The research institution’s Institutional Animal Care and Use Committee approved the research protocol [6].

In the study conducted by Draeger, the third toe of female broiler chickens was locally injected, into the tendon sheath, with 0.3 mL of gentamicin-sensitive *Staphylococcus aureus*. Twenty-four hours following the inoculation, the tendons receiving the standard treatment were surgically drained while the steroid treated tendons were injected with 0.06 mg/kg of dexamethasone in the tendon sheath. Additional corticosteroid injections were given at two, four, seven and fourteen days following the initial treatment. All chickens received antibiotics twenty-four hours post-inoculation (5 mg/kg of gentamicin [Vedco]). The standard treatment group received systemic doses, delivered intramuscularly, immediately following surgical drainage, and continued to receive the drug systemically twice a day for five days. The steroid group received a single local injection of gentamicin, administered into the flexor tendon sheath followed by systemic injections of the antibiotic twice a day for five days [6].

The range of motion was then assessed over the course of twenty-eight days, post-treatment. Once all measurements and data were collected, the animals were euthanized. The limbs were dissected and preserved by freezing at −20°C [6]. Tissue stored in this fashion demonstrates no significant change in biomechanical properties [15].

Of the chicken feet from the standard treatment and the steroid groups tested by Draeger (twenty-nine in total), twenty-three were examined in the current study (fifteen and eight, respectively). Unfortunately, due to prolonged time between thawing and testing, the remaining six specimens were discarded.

The third toe of the chicken was used due to its length and tenosynovial sheath similarities to the human finger. The tendon examined in this study was the flexor profundus, the deepest of the three tendons housed in the chicken’s toe. The flexor profundus was used due to its additional length and location within the digit.

The feet were thawed over night in a refrigerator and then the tendons were harvested. The same investigator preformed all surgical dissections. During the removal, a mark was made on the profundus with a permanent marker (Sharpie, Oak Brook, IL) fifteen millimeters distally from the interdigital space. This mark was used later in the testing process as a placement marker. After harvest, the cross-sectional area was measured at the location of the placement marker using an area micrometer with 0.12MPa of compressive stress applied [16].

Tendons were vertically mounted onto a materials testing system (Instron 8500 Plus, Instron Corporation, Norwood, MA) by means of two cryoclamps. The tendon was placed so as to have the placement marker positioned in the middle of the forty-five millimeter space between the two clamps. The flow of liquid nitrogen through a chamber within the cryoclamp was used to freeze the tendon in the clamp to prevent slippage of the tendons.

A 500N load cell (MTS Systems Corporation, Eden Prairie, MN) was used and tendon deformation was measured by grip displacement. Tendons were stretched at a ramp rate of 12 millimeters per minute until failure. The load and deformation signals were digitally sampled at twenty points per second and transferred to a computer for analysis by testing software (Series IX, Instron Corporation, Norwood, MA). The following structural properties and their corresponding material properties were measured from the load-deformation data: ultimate load, stiffness between 25-75% of ultimate load, energy to ultimate load, and displacement to ultimate load.

Tendons were continuously moistened with saline throughout the dissection and testing procedures.

The measurements from each group were analyzed by routine descriptive statistics (mean, standard deviation) and compared by means of Student’s unpaired t-test, where an alpha value of 0.05 was considered significant.

A post-hoc power analysis test was performed for each measurement in order to assess the likelihood that we missed detecting a clinically significant difference. In performing this power analysis we specified a 20% difference between the means as being clinically significant, as this value is a generally accepted threshold in clinic.

## Results

As can be seen from Table 1, the data collected from this study demonstrated trends for the tendons from the steroid group to be weaker and less stiff than those tendons which received the current standard treatment. However, the differences did not prove to be statistically significant for either the material or the structural properties.

**Table 1:** Statistical summary of data

		**Load at Max Load (N)**	**Stiffness (N/mm)**	**Stress at Max Load (Mpa)**	**Elastic Modulus (Mpa)**	**Area (mm**^**2**^**)**
	Mean	313	118	117	876	2.7
**Standard treatment**	Standard Deviation	46	30	16	225	0.3
	95% Confidence Interval	(286–339)	(100–136)	(107–126)	(740–1012)	(2.6-2.9)
		**Load at Max Load (N)**	**Stiffness (N/mm)**	**Stress at Max Load (Mpa)**	**Elastic Modulus (Mpa)**	**Area (mm**^**2**^**)**
	Mean	267	110	106	890	2.5
**Steroid treated**	Standard Deviation	66	30	20	309	0.2
	95% Confidence Interval	(212–322)	(85–136)	(89–122)	(623–1149)	(2.4-2.7)

Table presenting statistical data from each group tested. Standard treatment = systemic antibiotics and surgical drainage without adjunct corticosteroids. Steroid treated = local antibiotics and local corticosteroid injection without surgical drainage.

The ultimate load of the steroid group was 15% less than the standard treatment group but this difference was not statistically significant (p = 0.070, power = 0.822). The steroid group was also less stiff than the standard treatment group, but again this was not a statistically significant difference (p = 0.595, power = 0.385).

The material properties, ultimate stress and elastic modulus, also demonstrated no significant difference between the steroid group and the standard treatment group (p = 0.169, power = 0.885 and p = 0.913, power = 0.377 respectively).

The steroid group had a lower cross sectional area than the standard treatment group but again, this difference was not statistically significant (p = 0.082, power = 0.989).

## Discussion

The study conducted by Draeger et al. demonstrated that the chicken tendons, suffering from PFT, treated with corticosteroid injections had a more rapid recovery of functional motion compared to tendons which received the current standard treatment. This biomechanical evaluation found reductions in tendon strength, stiffness and material properties to be less than 20% and not statistically significant, addressing the concerns expressed over the use of corticosteroids as an adjunct treatment in PFT.

There is evidence that indicates PFT behaves similarly to other closed-space infections and therefore it is plausible that this regimen may produce similar results in treating an analogous ailment [13,17-21].

Our results reflect that of other literature which present similar findings [11,22-24]. Mackie et al. reported a decrease in tendon weight and area, but no significant decrease in overall tendon tensile strength when ten 0.1 mg/kg betamethasone paratendon injections were administered to the extensor retinaculum of a normal rabbit paw [23]. A similar conclusion was drawn by Matthews and associates when tendon tensile strength was measured after three to four 0.1 ml methylprednisolone injections were administered into rabbit patellar tendon sheath [24].

A strength of this investigation was the ability to use the specimens from Draeger’s study, as it allowed us to directly correlate the findings from the current study to those of the previous one.

As with all animal studies, our investigation had some limitations. Not all of the tendons from the original Draeger et al. study were examined, as six specimens were discarded; however, there was no selection bias in this loss. Also, as these tendons were tested after being frozen at −20°C, their strength may have been affected. However, all groups were frozen and thawed in the same manner, making any change to the integrity of the tendons uniform across all groups.

Additionally, we cannot be sure that this treatment regimen would have the same results in human tendons, which are rarely seen with such controlled and localized infection as our study presents. Finally, there may be effects that did not become apparent during this relatively short 28 day evaluation.

## Conclusion

In conclusion, our data demonstrates that treatment involving local antibiotic and steroid injections without surgical drainage for PFT, which produced the best range of motion in the study by Draeger et al., does not lead to a statistically significant loss of tendon strength when compared to tendons administered with the current standard treatment. It must be stressed that this study investigated the effects after a four-week recovery period and that clinical trials are warranted to determine whether or not this specific treatment should be considered as a treatment regimen for patients suffering from PFT.

## Abbreviations

PFT: Pyogenic flexor tenosynovitis.

## Competing interests

No competing interests arose during this investigation.

## Authors’ contributions

BT dissected and tested the specimens, as well as interpreted the data and drafted the manuscript. PW supervised the testing. RD performed the original surgeries and measurements. PW, RD, DB, and LD conceived of the study and its design and also helped in drafting the manuscript. All authors read and approved the final manuscript.

## Authors’ information

BT is a medical student. PW is an associate research professor of orthopaedics at the research institution. RD is a 4^th^ year orthopaedic resident at the research institution. Both DB and LD are clinical surgeons and professors of orthopaedics at the research institution.
